# The Information Sharing Behaviors of Dietitians and Twitter Users in the Nutrition and COVID-19 Infodemic: Content Analysis Study of Tweets

**DOI:** 10.2196/38573

**Published:** 2022-09-16

**Authors:** Esther Charbonneau, Sehl Mellouli, Arbi Chouikh, Laurie-Jane Couture, Sophie Desroches

**Affiliations:** 1 Centre Nutrition, Santé et Société Institute of Nutrition and Functional Foods Université Laval Quebec City, QC Canada; 2 School of Nutrition Université Laval Quebec City, QC Canada; 3 Faculty of Business Administration Université Laval Quebec City, QC Canada

**Keywords:** nutrition, COVID-19, dietitians, Twitter, public, themes, behavior, content accuracy, user engagement, content analysis, misinformation, disinformation, infodemic

## Abstract

**Background:**

The COVID-19 pandemic has generated an infodemic, an overabundance of online and offline information. In this context, accurate information as well as misinformation and disinformation about the links between nutrition and COVID-19 have circulated on Twitter since the onset of the pandemic.

**Objective:**

The purpose of this study was to compare tweets on nutrition in times of COVID-19 published by 2 groups, namely, a preidentified group of dietitians and a group of general users of Twitter, in terms of themes, content accuracy, use of behavior change factors, and user engagement, in order to contrast their information sharing behaviors during the pandemic.

**Methods:**

Public English-language tweets published between December 31, 2019, and December 31, 2020, by 625 dietitians from Canada and the United States, and Twitter users were collected using hashtags and keywords related to nutrition and COVID-19. After filtration, tweets were coded against an original codebook of themes and the Theoretical Domains Framework (TDF) for identifying behavior change factors, and were compared to reliable nutritional recommendations pertaining to COVID-19. The numbers of likes, replies, and retweets per tweet were also collected to determine user engagement.

**Results:**

In total, 2886 tweets (dietitians, n=1417; public, n=1469) were included in the analyses. Differences in frequency between groups were found in 11 out of 15 themes. Grocery (271/1417, 19.1%), and diets and dietary patterns (n=507, 34.5%) were the most frequently addressed themes by dietitians and the public, respectively. For 9 out of 14 TDF domains, there were differences in the frequency of usage between groups. “Skills” was the most used domain by both groups, although they used it in different proportions (dietitians: 612/1417, 43.2% vs public: 529/1469, 36.0%; *P*<.001). A higher proportion of dietitians’ tweets were accurate compared with the public’s tweets (532/575, 92.5% vs 250/382, 65.5%; *P*<.001). The results for user engagement were mixed. While engagement by likes varied between groups according to the theme, engagement by replies and retweets was similar across themes but varied according to the group.

**Conclusions:**

Differences in tweets between groups, notably ones related to content accuracy, themes, and engagement in the form of likes, shed light on potentially useful and relevant elements to include in timely social media interventions aiming at fighting the COVID-19–related infodemic or future infodemics.

## Introduction

### Background

On January 7, 2020, Chinese health authorities officially announced the emergence of the disease caused by the 2019 novel coronavirus [1] or SARS-CoV-2, a new strain of coronavirus [2]. COVID-19 was then declared a pandemic by the World Health Organization on March 11, 2020 [3], and as of March 24, 2022, the infection resulted in 470,223,960 confirmed cases and 6,094,326 deaths worldwide [4]. COVID-19 is characterized by symptoms ranging from cough and fever [5] to severe pneumonia and central nervous system damage [6]. Besides potential long-term health consequences with long COVID-19 [7], the infection led to serious social and economic repercussions [8]. To date, COVID-19 vaccines are the only man-made product (as opposed to infection-induced immunity) able to build one’s immunity against SARS-CoV-2 [9].

From its onset, the pandemic has triggered multiple studies as clinical data were rapidly needed to face and fight the infection [10]. One area of study that retained researcher attention was related to the link between COVID-19 and nutrition. Indeed, concerns have been raised about certain nutrition-related health conditions, namely, diabetes, obesity, and cardiovascular diseases, as these could potentially elevate one’s risk of experiencing severe COVID-19 [11]. Moreover, the roles played by nutrients, foods, and other types of supplements in immunity and inflammation have been studied extensively. For instance, Iddir et al [12] studied the role of certain nutrients and phytochemicals in reducing oxidative stress and inflammation, and underlined the importance of an optimal nutritional status in immunity. Furthermore, the pandemic has given rise to food- and nutrition-related changes in individuals, including those pertaining to food security [13], weight [14], and food habits [15]. These new data have led health organizations to develop recommendations and guidelines with regard to the appropriate food habits and nutritional care to follow during the COVID-19 pandemic [16,17].

In parallel, social media are equally being used as sources of health information and as platforms to disseminate health-related recommendations [18]. More specifically, Twitter, a microblogging site that permits real-time communication of 280-character tweets with followers [19], is considered a useful public health tool to share health-related information and engage with the public. As a matter of fact, it has been used by health professionals to provide information, educate people, share updates, disseminate new research, and raise public awareness of health matters like nutrition, infectious diseases, and sanitary emergencies [20,21]. However, concerns have been raised regarding the reliability and accuracy of the information found on social media such as Twitter [18].

Indeed, recently, the COVID-19 pandemic has played a major role in demonstrating how social media can be helpful as well as detrimental. The pandemic has led to what the World Health Organization calls an “infodemic,” an overabundance of information online and offline, which may be true or false. Although an infodemic is not solely characterized by false information, it certainly contributes to its propagation. This situation can result in different repercussions, including damage to physical and mental health, increased stigma and conflict, and a lack of compliance with public health measures [22]. Moreover, at the beginning of the pandemic, Twitter was criticized, as most of the false information circulating on the platform was not verified [23]. However, efforts have been made by the microblogging service to counter misleading information [24]. Before going further, the types of false information should be distinguished. False information includes both misinformation and disinformation. Although the former is unintentional, the latter is done deliberately, in order to cause harm [25]. Both terms will be used jointly in this paper, as it can be hypothesized that both take place, but it is not part of the objectives of this study to determine the intent behind false information sharing.

Nutrition has received interest from researchers, official health organizations, and the general population since the beginning of the pandemic. In parallel, social media posts to this effect have also risen, and it is possible that misinformation and disinformation have also reached some of these communication platforms. Knowing this, some sources of information, including lay people, can be unreliable and could contribute to the proliferation and dissemination of misinformation and disinformation on nutrition-related topics. Conversely, dietitians are recognized as nutrition experts and should be prioritized when seeking information on food and nutrition [16]. A comparison between dietitians and general Twitter users relative to nutrition-related tweets has the potential to support the need for exercising caution when using Twitter, given the infodemic and the presence of unverified information on the microblogging site at the start of the pandemic [22,23], as well as for emphasizing the important role of dietitians on social media. Additionally, to our knowledge, only a few studies have documented misinformation or disinformation related to nutrition and COVID-19 altogether. These studies were however focused on specific aspects of nutrition such as immunity boosting claims [9,26].

### Influence of Social Media on Behavior

Researchers have started exploring how social media publications regarding COVID-19 could influence intention, behavior, and protection against the virus [27-29]. More specifically, Al-Dmour et al showed that the use of social media platforms, including Facebook, Instagram, and Twitter, results in public protection from the infection, through the mediating effects of public health awareness and public health behavioral changes [29]. These results support the potential influence of social media publications over users. Nonetheless, food- and nutrition-related behaviors have not been investigated in that sense. Moreover, given that misinformation and disinformation can be found in tweets, it is important to explore the factors used, intentionally or not, in publications, as they could potentially influence behavior. To this end, behavior change theoretical models can be useful to highlight such factors, as well as to understand behavior change. Such models have also been used to build social media interventions aiming to modify health-related behaviors like vaccination [30]. One of these models, the Theoretical Domains Framework (TDF), was initially developed to resolve the issue of having an overabundance of theoretical models and constructs aiming to explain behavior change [31], and is most often being used in implementation research in an array of settings, including health care, namely to identify the facilitators and barriers to implementing evidence-based behaviors or to design interventions [32]. Recently, the TDF has also been applied to content analysis of social media publications to determine the factors explaining COVID-19 vaccine hesitancy [33], thus providing a strategy to explore the behavior change potential of social media.

### Objectives and Research Questions

The aim of this study was to compare the information sharing behaviors of registered dietitians (RDs) and Twitter users during the infodemic by analyzing their tweets related to nutrition in times of COVID-19. To do so, we compared the tweets of the 2 groups in terms of their themes, the user engagement they generated, content accuracy, and whether tweets included behavior change factors. To this end, we elaborated some research questions to be answered. Research questions are normally inquisitive in nature and better suited for exploratory studies where too little data are available to develop hypotheses [34], as in this study. The research questions were as follows:

1. What are the differences between dietitians’ tweets and the public’s tweets in terms of the themes they discuss?

2. What are the differences between dietitians’ tweets and the public’s tweets in terms of the engagement they receive from users?

3. What is the difference in content accuracy between dietitians’ tweets and the public’s tweets?

4. What are the differences between dietitians’ tweets and the public’s tweets in terms of the TDF domains they use, and could their tweets influence behavior?

## Methods

### Overview

**Figure 1:**
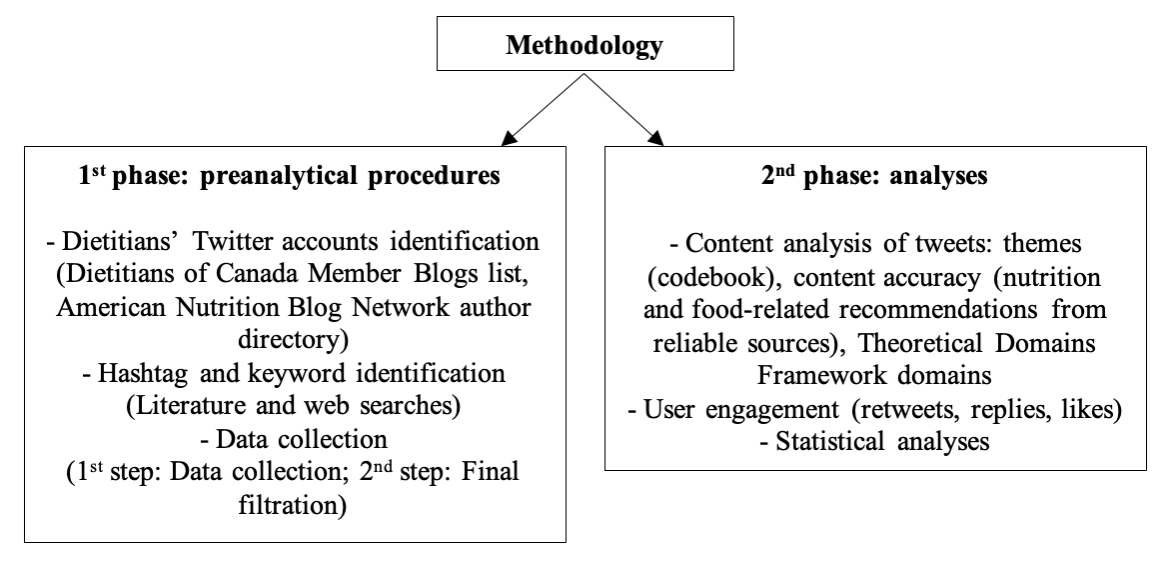
Study’s synthesized methodology.

This study’s methods can be divided in 2 phases, namely, preanalytical procedures and analyses, as represented in Figure 1.

### Dietitians’ Twitter Account Identification

**Figure 2:**
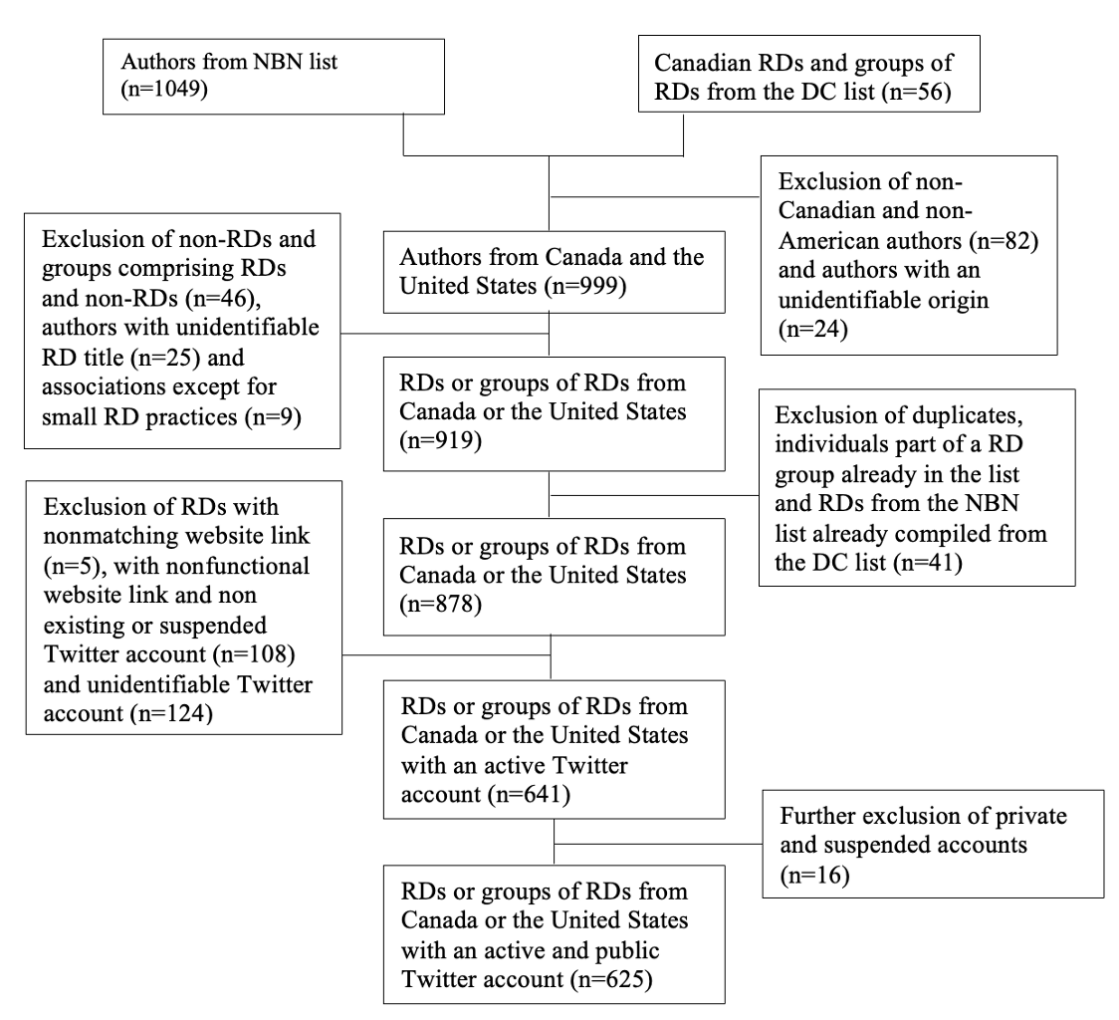
Flow chart detailing the steps for creating the registered dietitians (RDs) list using the Nutrition Blog Network (NBN) author directory and the Dietitians of Canada (DC) Member Blogs list.

In order to identify our sample of RDs from Canada and the United States with Twitter accounts, the Dietitians of Canada Member Blogs list [35] (n=56 as of October 2020) and the American Nutrition Blog Network author directory [36] (n=1049 as of October 2020) were used. Both directories were reviewed to create a list of RDs (n=641), which included their name, website title, and Twitter handle. From this list, 16 RDs were excluded owing to suspended or private accounts. The final list thus comprised a total of 625 Twitter accounts. The steps are detailed in Figure 2.

### Hashtag and Keyword Identification

A predetermined list of 2561 hashtags and keywords related to COVID-19, 41 hashtags related to nutrition, and 16 hashtags related to both was used to filter tweets from the public and RDs (eg, “coronavirus,” “#immunity,” “#coviddiet,” “#health,” and “#nutrition”). The method for identifying hashtags and keywords was inspired by previous studies [37-39]. First, the list was built based upon searches on Facebook, Instagram, and Twitter of hashtags and keywords relevant to COVID-19, nutrition, or both. Second, it was enriched through literature [39-45] and web searches [46-49]. Two websites, Tagdef [46] and besthashtags [47], are generally used to find currently trending hashtags. The terms “COVID and nutrition,” “COVID-19 and nutrition,” “coronavirus and nutrition,” and “corona and nutrition” were used to obtain hashtags related to these topics. Moreover, the literature was searched with terms related to nutrition, COVID-19, and Twitter to find studies containing relevant hashtags. Finally, we verified each keyword and hashtag to ensure its relevance.

### Data Collection

To be considered for the analysis, tweets had to be written in English, discuss at least one aspect of nutrition in times of COVID-19, and be published between December 31, 2019, and December 31, 2020. December 31, 2019, marks the date when cases of an unknown acute respiratory disease in Wuhan were first reported by Chinese health authorities [1]. Conversely, publications containing no written content or link to supplementary information were excluded. Tweets were collected in 2 steps using the Twitter Premium Application Programming Interface (API), which permits access to the Twitter archive. The publication date, author name, description, and country of origin (when available), as well as the numbers of likes, replies, and retweets were collected. Moreover, tweets from Twitter users were filtered to avoid having RDs from our list in that subsample.

**Figure 3:**
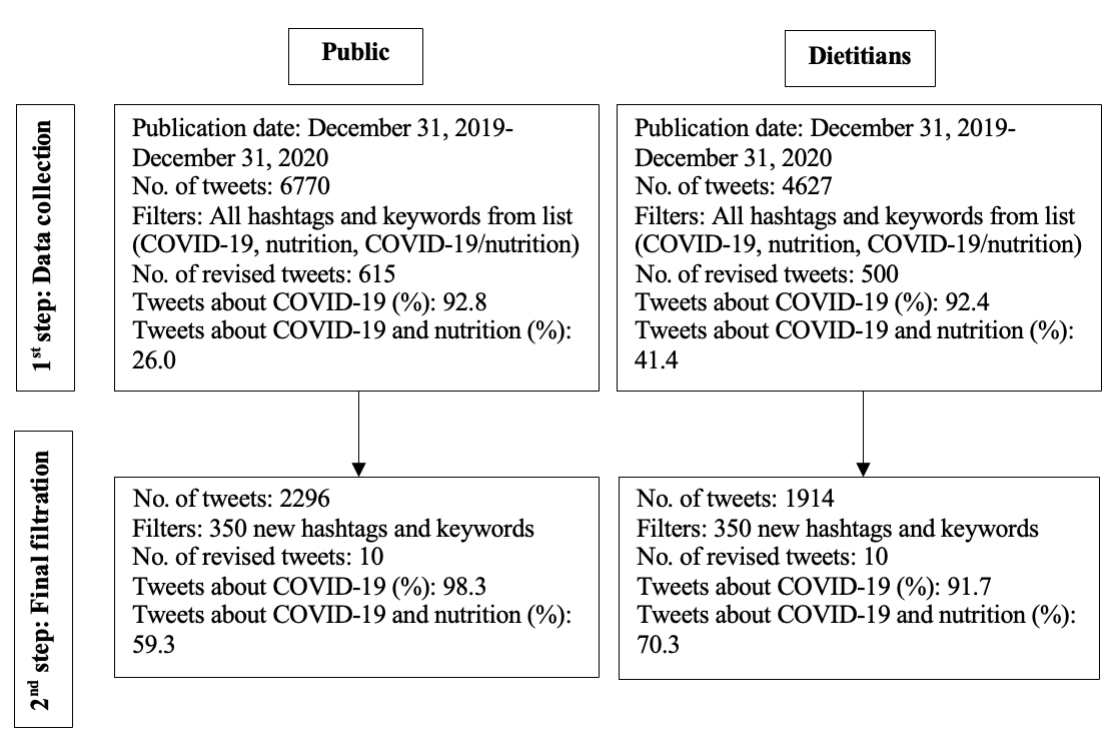
Steps detailing tweet collection resulting in the final samples for analysis.

Thus, the first step consisted of collecting the data using a predetermined list of hashtags and keywords, which resulted in 6670 tweets for the public group and 4627 tweets for the dietitian group. After revising a subsample of each group, we observed that only 26.0% and 41.4% of the public’s tweets and dietitians’ tweets, respectively, were about both nutrition and COVID-19. The predetermined list of hashtags and keywords was thus enriched to render our data more specific to COVID-19 and nutrition. First, using tweets pertaining to COVID-19/nutrition from our 2 revised subsamples (see step 1 in Figure 3), 2 coders noted all the hashtags and keywords about nutrition and COVID-19/nutrition that were not already in our predetermined list (eg, #weightloss and #COVIDbaking). Second, these were compiled in a new list of 332 hashtags and keywords referring to nutrition and another 18 referring to COVID-19 and nutrition. Then, in the second step, the public and dietitian samples were submitted to a final filtration using this list of 350 hashtags and keywords. This process allowed the generation of 2 samples more specific to COVID-19 and nutrition. The steps are detailed in Figure 3.

Considering the difficulties associated with this type of data collection [50] and that it was important not to reduce data representativeness, it was agreed not to further iterate the data sets. The final sample thus included a total of 4210 tweets (RDs: n=1914; public: n=2296). These 2296 tweets from the public were published by 1043 users. During coding, tweets that were still not related to COVID-19 and nutrition altogether were documented but not analyzed. Often, this happened when tweets contained hashtags or keywords about COVID-19 but not about nutrition specifically as in the following case: “[…] #needsofchildren #artathome #StatHomeStayCreative #coronavirustips.” Hence, out of the 1914 tweets in the dietitian group, 1417 were included in the final analyses described below. As for the public group, 1469 out of 2296 tweets were analyzed. Thus, there were a total of 2886 tweets in both groups. When associated content was available through links in the tweet, it was also coded as part of the tweet.

### Analyses

#### Research Question #1: Themes

The infodemic has generated multiple discussions on social media, which can reduce access to reliable information [51]. Defining the themes discussed about nutrition and COVID-19 on Twitter helps in determining which of them need to be more or less addressed by reliable sources of information on nutrition. Themes are patterns of information that represent categories to be analyzed [52]. This analysis was conducted to determine what subjects (RDs and the public) discuss with regard to the nutrition and COVID-19–related infodemic. Coders followed an iterative process based on the methodologies of similar studies to inductively create a codebook of themes [53,54]. First, 2 team members (EC and LJC) each elaborated a list of themes based on a review of the same 100 tweets published by Twitter users from the public. Second, common themes were put together to create an initial codebook. Third, since there were discrepancies between coders, each reviewed the same 50 tweets published by RDs, which led to the improvement of the initial codebook. Fourth, themes for which there was still no agreement were settled by SD. Then, a codebook comprising 16 themes was established. Fifth, after the first round of reliability coding, the theme “Stress and Anxiety” was eliminated and added to the theme changed from “Physical Activity” to “Other Lifestyle Habits.” It was thought relevant to address COVID-19–related lifestyle habits, but it was decided to regroup them into a single theme as they were not specific to nutrition. A final codebook including 15 nonmutually exclusive themes was then established and used by the same 2 investigators to categorize the 2886 tweets. Saturation, which was determined by identifying the point where all themes had been addressed at least once, was reached after 105 tweets for the dietitian group and 71 tweets for the public group. The theme frequency was compared between groups. Based on the tweet publication date, the frequency was also determined in the first 2 waves of the pandemic (first: December 31, 2019, to July 31, 2020; second: August 1, 2020, to December 31, 2020) and compared between waves. To render a more precise description and comparison of themes, statistics were used to compare theme frequencies between groups.

#### Research Question #2: User Engagement

Members of the public are not necessarily reliable sources of information on nutrition, while dietitians are considered reliable sources. This can become problematic when members of the public generate more engagement in their posts than their expert counterparts. In order to find out whether certain themes were more popular than others from a reader’s perspective, the user engagement generated by themes was evaluated based on the numbers of likes, replies, and retweets associated with tweets. More specifically, for both subsamples separately, the mean numbers of likes, replies, and retweets for a single tweet were calculated for each theme. The means were then compared between groups to determine if certain themes were more popular in one group than the other. Additionally, the proportion of dietitians’ tweets related to COVID-19 and nutrition out of their total yearly publications was calculated to evaluate their own engagement in this conversation on Twitter.

#### Research Question #3: Content Accuracy

To determine tweets’ content accuracy and thus reveal the presence of misinformation, 2 team members (EC and LJC) compared the 2886 tweets against evidence-based nutrition and food-related recommendations regarding COVID-19. First, a database of recommendations from reliable and expert sources that covered COVID-19 and nutrition-related themes, such as Dietitians of Canada, Health Canada, and the Academy of Nutrition and Dietetics, was elaborated through web searches. However, when a tweet’s content was too specific to be compared to the aforementioned recommendations, it became necessary to use more specialized sources of information (eg, PubMed and Mayo Clinic). For instance, the following tweet’s content could not be found in our database of recommendations: “If your body happens to change during the pandemic, it could be because of stress […].” Second, during coding, coders read the tweet and verified its information using one or many reliable recommendations pertaining to the specific content of that tweet. If its content was in line with the recommendation, it was deemed accurate. If the content differed from the recommendation in any way, it was deemed inaccurate. Thus, tweets were categorized as accurate, inaccurate, or not applicable. The “not applicable” category was used when it was impossible to determine the tweet’s accuracy for one or more of the following reasons: (1) the tweet is sharing a recipe or meal idea, (2) it is formulated as a question, (3) it reports on study results, and (4) it is considered as a nonscientific declaration or an opinion. For this study, it was decided that although study results pertaining to COVID-19 and nutrition could be compared to other studies, which are part of a body of evidence still in development, they include emerging data and not suggestions or advice to be followed. Moreover, they are too preliminary and specific to their study’s methodology and population to be compared against nutritional recommendations about COVID-19. Moreover, although opinions or nonscientific declarations can be based on unsupported claims, for this study, it was decided that they could not be evaluated for accuracy. Indeed, this category could include tweets related to, for instance, what the users ate that day, a new nutrition-related habit they developed during the pandemic, or words of encouragement for workers in the food industry. As the evaluation went on from April through July 2021 and was then based on the current and available recommendations at that time, it is possible that the categorization would be different at the time when this paper has been written or published. Nevertheless, we made sure to use the most up to date information by regularly verifying updates in recommendations and available documentation. Saturation, which was determined by identifying the point where the 3 possible categorizations had been coded at least once, was reached after 25 tweets for the dietitian group and 13 tweets for the public group. Finally, the frequencies of accurate and inaccurate tweets were compared between groups. The frequencies of the nonapplicable categorization and of the 4 reasons why a tweet’s accuracy could not be evaluated were also compared between groups. Moreover, further analyses were performed to compare the numbers of accurate and inaccurate mentions for each theme, so as to bring out those more frequently inaccurate than accurate.

#### Research Question #4: TDF Domains

Acting upon misinformation and disinformation can have detrimental effects. Therefore, to verify if tweets could potentially influence readers’ behaviors, the 2886 tweets were deductively coded by 2 team members (EC and LJC) using the second version of the TDF [32]. The TDF does not serve as the theoretical lens for the whole study but solely to conduct an analysis aiming to determine whether tweets carry factors that could influence individual behavior. To our knowledge, the TDF has only been applied once before to tweets [33] and is thus a new application to be explored. Table 1 presents the 14 domains reflecting the cognitive, affective, social, and environmental factors influencing behaviors and their descriptions. This behavior change framework was chosen because it facilitates categorization during coding, as distinctive domains can be identified within tweets. Moreover, the TDF is highly comprehensive as it is based upon 33 theories and 128 theoretical constructs related to behavior change [55]. Thus, this model is useful to analyze a wide range of behaviors, which is the case in this study.

**Table 1:** Description of the Theoretical Domains Framework domains.

Domain	Description [32]
Knowledge	Awareness of something
Skills	Ability or competence developed through practice
Social and professional role and identity	Individual behaviors and qualities displayed in a social or work setting
Beliefs about capabilities	Recognition of one’s competences and abilities that can be put to constructive use
Optimism	Confidence that goals and desires will be reached
Beliefs about consequences	Expectancies about outcomes of a behavior in a situation
Reinforcement	Increasing the probability of a behavior with a stimulus
Intentions	Decision to accomplish a behavior or to act in a certain way
Goals	Mental representations of outcomes one wants to attain
Memory, attention, and decision processes	Ability to remember information, focus, and choose between different alternatives
Environmental context and resources	Situational or environmental aspect of one’s life that encourages or discourages the adoption of an adaptive behavior, skill, or competence
Social influences	Interpersonal processes that lead one to modify their thoughts, feelings, or behaviors
Emotion	Complex reaction by which one attempts to manage a personally significant matter or event
Behavioral regulation	Something done to manage or change one’s actions

The 14 domains were not mutually exclusive. Saturation, which was determined by identifying the point where all domains had been addressed at least once, was reached after 54 tweets for the dietitian group and 13 tweets for the public group. The frequency of each domain was compared between groups. Exploratory analyses were also conducted to reveal the most and least frequent domains for each theme.

Lastly, intercoder agreement, which measures the degree of similarity in codes assigned to a data set by different coders, was determined so as to preserve the consistency of results during individual coding [56]. Thus, the first round of reliability coding was performed where the 2 coders (EC and LJC) analyzed 100 tweets from each group according to the 3 content analyses described above, after which coders met to establish consensus. As scores for some themes and domains were too low, a second round of reliability coding was completed where both coders each analyzed 50 tweets from each group and met again to establish consensus. As scores obtained for themes and domains were satisfying, it was agreed that coding could be initiated. The kappa scores are presented in Table 2. Kappa scores ranging from 0.61 to 0.80 demonstrate substantial agreement between coders, while scores ranging from 0.81 to 1.00 are interpreted as almost perfect agreement [57]. For the rest of the sample, both team members coded 850 and 914 tweets from the dietitian group, respectively, which included 1 round of reliability coding of 100 tweets. They also coded 1000 and 1146 tweets from the general public group, respectively, including 2 rounds of reliability coding of 100 tweets each.

**Table 2:** Kappa scores obtained after 2 rounds of reliability coding.

Group	COVID-19/nutrition or not (1st round)	Content accuracy (1st round)	Themes (1st and 2nd rounds)	Domains (1st and 2nd rounds)
Public	0.78	0.67	0.54 and 0.65	0.42 and 0.63
Dietitian	0.95	0.78	0.51 and 0.79	0.66 and 0.75

### Statistical Analyses

Statistical analyses were performed in SAS OnDemand for Academics (SAS Institute Inc). A *P* value ≤.05 (2 sided) was considered significant. This level of significance is often chosen in research [58]. The *P* value is the probability that measures the likeliness of a difference between groups being due to chance [59]. Chi-square tests were used to compare theme frequencies between groups and between the 2 waves of the pandemic. Chi-square tests were also used to compare the frequencies of the TDF domains, accurate/inaccurate categorization, and reasons for nonapplicability between groups. Comparisons of the frequencies of inaccurate and accurate mentions for each theme were also conducted using the chi-square test. The Fisher exact test was used instead of the chi-square test when at least one cell contained less than 5 data points [60]. Differences in means of likes, replies, and retweets per tweet between dietitians and the public were assessed by the nonparametric version of the *t* test for continuous data, that is, the Wilcoxon rank-sum test, as the data were not normally distributed and samples were independent [61,62].

### Ethical Considerations

The Université Laval Research Ethics Board exempted this project from ethical review as analyses were completed with publicly available content. However, complete examples of tweets have not been presented in order to preserve the anonymity of the Twitter users.

## Results

### Research Question #1: Themes

The number of themes about nutrition and COVID-19 found in this study supports the fact that the infodemic has also reached this thematic. Table 3 shows the number of times each theme was addressed by both groups. In our sample, grocery, and diets and dietary patterns were the most frequently discussed themes by dietitians (271/1417, 19.1%) and the public (507/1469, 34.5%), respectively. Furthermore, many differences were found between the groups. For instance, weight loss was a more frequently discussed theme among the public than among dietitians (106/1469, 7.2% vs 24/1417, 1.7%; *P*<.001). Conversely, immune health was more frequently addressed by dietitians than by the public (177/1417, 12.5% vs 87/1469, 5.9%; *P*<.001).

**Table 3:** Comparison of theme frequencies between groups.

Theme	Description	Dietitian group (N=1417), n (%)	Public group (N=1469), n (%)	*P* value
Weight loss	Tips, mention, desire, and promotion. Not necessarily due to the pandemic.	24 (1.7)	106 (7.2)	<.001
Cooking and recipes	Sharing of recipes or meal/snack ideas. Mentions of what the next meal will be.	215 (15.2)	214 (14.6)	.65
Immune health	Linking nutrients, supplements, and foods, as well as physical activity, healthy eating, and hydration with immunity.	177 (12.5)	87 (5.9)	<.001
Food support and food system	Food support programs, food services/systems, buying local, gardening, and food insecurity.	206 (14.5)	59 (4.0)	<.001
Specific foods	Mention, consumption, or promotion of foods of various nutritional values.	178 (12.6)	487 (33.2)	<.001
Alcohol consumption	Reference to alcohol or mention of consumption.	19 (1.3)	86 (5.9)	<.001
Nutrients and supplements	Mention or promotion of a nutrient or supplement, regardless of immunity.	80 (5.7)	81 (5.5)	.88
Overeating	Mention of eating a large quantity of food in one sitting.	18 (1.3)	65 (4.4)	<.001
Food tips and recommendations	Hydration, suggestion of certain foods or practices, healthy restaurant food choices, and sanitary measures in restaurants.	253 (17.9)	108 (7.4)	<.001
Food changes	Modification of food choices, habits, and offers due to the pandemic, except for diets.	173 (12.2)	149 (10.1)	.08
Body appearance	References to physical appearance regardless of weight loss; includes weight gain.	86 (6.1)	67 (4.6)	.07
Diets and dietary patterns	Mention or promotion of diets, dietary patterns, and related practices.	26 (1.8)	507 (34.5)	<.001
Other lifestyle habits	References to physical activity (without mention of weight loss), stress/anxiety, sleep, tobacco, and cannabis.	259 (18.3)	453 (30.8)	<.001
Grocery	Food safety, in-store sanitary measures, healthy food choices at the store, ways to reduce grocery bills, and increased/decreased availability of products.	271 (19.1)	68 (4.6)	<.001
Health care system	Changes in dietetics practice, underlying health conditions, and nutrition of infected patients.	209 (14.8)	23 (1.6)	<.001

Comparison of themes between the first 2 waves of the pandemic revealed that none of the themes were more frequently addressed in the second wave than in the first by either of the groups. Indeed, 83.0% of dietitians’ tweets were published during the first wave. Weight loss (*P*=.03), cooking and recipes (*P*<.001), specific foods (*P*=.03), food tips and recommendations (*P*=.003), grocery (*P*<.001), and health care system (*P*<.001) were more frequently addressed in the first wave than in the second. As for the public, they published 93.7% of their tweets during the first wave. Food tips and recommendations (*P*<.001), physical appearance (*P*=.02), and diets and dietary patterns (*P*=.03) were more frequent in the first wave than in the second wave. These results indicate that the first wave generally led to more discussions than the second wave.

### Research Question #2: Social Media Engagement

Tables 4-6 show the comparisons of the mean numbers of retweets, replies, and likes per tweet for each theme between groups. The results revealed that dietitians constantly received a higher number of retweets per tweet than the public. Conversely, the public had more replies per tweet than dietitians. However, the public rarely had more than one reply per tweet, indicating that replies were seldom used by readers to manifest their engagement in both groups. Furthermore, while engagement by replies and retweets depended on the group rather than the theme, engagement by likes varied between groups according to the theme. Indeed, weight loss, immune health, food support and food system, nutrients and supplements, and food tips and recommendations were more popular when addressed by dietitians, as other lifestyle habits generated more interest in the public’s tweets. Moreover, it was observed that out of 73,323 English-language tweets published by dietitians during the 1-year period, only 1417 (1.9%) pertained to COVID-19 and nutrition. Lastly, there was no difference in the number of followers between groups. In the dietitian group, retweet and follower counts were not associated (*r*=0.04; *P*=.16), while there was an association between like and follower counts (*r*=0.12; *P*<.001).

**Table 4:** Comparison of the mean number of retweets per tweet between groups.

Theme	Dietitian group		Public group		*P* value
	Number of tweets	Number of retweets per tweet, mean (SD)	Number of tweets	Number of retweets per tweet, mean (SD)	
Weight loss	24	23.96 (77.49)	106	0.066 (0.42)	<.001
Cooking and recipes	215	149.91 (2181.49)	214	0.028 (0.17)	<.001
Immune health	177	11.99 (65.36)	87	0.092 (0.33)	<.001
Food support and food system	206	569.18 (5040.55)	59	0.017 (0.13)	<.001
Specific foods	178	182.53 (23.97)	487	0.037 (0.22)	<.001
Alcohol consumption	19	76.16 (327.37)	86	0.047 (0.21)	<.001
Nutrients and supplements	80	9.61 (43.43)	81	0.11 (0.39)	<.001
Food overconsumption	18	7.78 (23.37)	65	0.015 (0.12)	<.001
Food tips and recommendations	253	45.71 (677.18)	108	0.14 (0.50)	<.001
Food changes	173	1197.90 (15693.72)	149	0.013 (0.12)	<.001
Body appearance	86	242.36 (1424.06)	67	0.045 (0.21)	<.001
Diets and dietary patterns	26	5.31 (15.71)	507	0.018 (0.15)	<.001
Other lifestyle habits	259	22.37 (141.21)	453	0.15 (0.67)	<.001
Grocery	271	1176.28 (9564.04)	68	0.074 (0.31)	<.001
Health care system	209	65.30 (624.76)	23	0 (0)	<.001

**Table 5:** Comparison of the mean number of replies per tweet between groups.

Theme	Dietitian group			Public group			*P* value
	Number of tweets	Number of replies per tweet, mean (SD)	Number of tweets		Number of replies per tweet, mean (SD)		
Weight loss	24	0 (0)	106		0.75 (5.073)	.02	
Cooking and recipes	215	0 (0)	214		0.32 (0.99)	<.001	
Immune health	177	0 (0)	87		0.023 (0.15)	.04	
Food support and food system	206	0 (0)	59		0.068 (0.31)	.001	
Specific foods	178	0 (0)	487		0.44 (1.15)	<.001	
Alcohol consumption	19	0 (0)	86		0.52 (1.49)	.01	
Nutrients and supplements	80	0 (0)	81		0.21 (1.03)	.003	
Food overconsumption	18	0 (0)	65		0.74 (1.57)	.01	
Food tips and recommendations	253	0 (0)	108		0.13 (0.91)	.002	
Food changes	173	0 (0)	149		0.66 (1.43)	<.001	
Body appearance	86	0 (0)	67		1.06 (6.37)	<.001	
Diets and dietary patterns	26	0 (0)	507		0.55 (2.57)	.005	
Other lifestyle habits	259	0 (0)	453		0.080 (0.34)	<.001	
Grocery	271	0 (0)	68		0.13 (0.39)	<.001	
Health care system	209	0 (0)	23		0.044 (0.21)	.003	

**Table 6:** Comparison of the mean number of likes per tweet between groups.

Theme	Dietitian group		Public group		*P* value
	Number of tweets	Number of likes per tweet, mean (SD)	Number of tweets	Number of likes per tweet, mean (SD)	
Weight loss	24	14.58 (32.60)	106	1.44 (3.73)	.03
Cooking and recipes	215	2.44 (5.88)	214	2.02 (5.20)	.18
Immune health	177	5.23 (30.95)	87	0.29 (0.59)	<.001
Food support and food system	206	2.67 (6.67)	59	0.41 (1.04)	.007
Specific foods	178	2.76 (7.66)	487	2.04 (4.72)	.65
Alcohol consumption	19	4.84 (13.87)	86	2.21 (5.37)	.66
Nutrients and supplements	80	2.00 (5.53)	81	1.02 (4.83)	.003
Food overconsumption	18	1.61 (2.48)	65	3.03 (6.22)	.51
Food tips and recommendations	253	1.66 (5.51)	108	0.69 (2.98)	<.001
Food changes	173	1.67 (2.90)	149	2.40 (5.30)	.46
Body appearance	86	5.70 (18.49)	67	1.78 (4.70)	.81
Diets and dietary patterns	26	13.85 (66.33)	507	2.11 (4.85)	.52
Other lifestyle habits	259	1.57 (4.23)	453	1.63 (11.74)	.04
Grocery	271	1.89 (6.06)	68	0.92 (1.66)	.34
Health care system	209	2.13 (8.89)	23	0.22 (0.42)	.06

### Research Question #3: Content Accuracy

Content accuracy analyses revealed the presence of misinformation, but mostly in the public’s tweets. In fact, a higher proportion of dietitians’ tweets were accurate compared with the public’s tweets (*P*<.001). For dietitians, out of a total of 575 tweets for which accuracy could be evaluated, 532 (92.5%) were accurate. As for the public, out of 382 tweets, 250 (65.5%) were accurate. Table 7 shows the comparison of the number of accurate and inaccurate tweets per theme. Weight loss was considered problematic as it had more inaccurate than accurate tweets. All other differences were in favor of accuracy.

**Table 7:** Content accuracy of individual themes.

Theme	Accurate (N=782), n (%)	Inaccurate (N=175), n (%)	*P* value
Weight loss	11 (1.4)	30 (17.1)	<.001
Cooking and recipes	45 (5.8)	15 (8.6)	.16
Immune health	128 (16.4)	77 (44.0)	<.001
Food support and food system	91 (11.6)	1 (0.6)	<.001
Specific foods	105 (13.4)	28 (16.0)	.37
Alcohol consumption	20 (2.6)	7 (4.0)	.30
Nutrients and supplements	78 (10.0)	26 (14.9)	.06
Food overconsumption	12 (1.5)	4 (2.3)	.51
Food tips and recommendations	224 (28.6)	17 (9.7)	<.001
Food changes	57 (7.3)	4 (2.3)	.02
Body appearance	14 (1.8)	3 (1.7)	<.99
Diets and dietary patterns	35 (4.5)	22 (12.6)	<.001
Other lifestyle habits	219 (28.0)	34 (19.4)	.02
Grocery	215 (27.5)	14 (8.0)	<.001
Health care system	74 (9.5)	5 (2.9)	.004

Furthermore, 842 (59.4%) of the dietitians’ tweets and 1087 (74.0%) of the public’s tweets were deemed not applicable for accuracy evaluation. More specifically, there were differences between groups for 3 reasons out of 4. First, a recipe or meal idea was shared more often in the public’s tweets than in dietitians’ tweets (332/1087, 30.5% vs 205/842, 24.4%; *P*=.003). Second, no difference was found between groups when tweets were formulated as a question. Third, study results were more frequently reported in dietitians’ tweets than in the public’s tweets (118/842, 14.0% vs 8/1087, 0.7%; *P*<.001). Fourth, opinions or nonscientific declarations were more frequently shared in the public’s tweets than in dietitians’ tweets (806/1087, 74.2% vs 551/842, 65.4%; *P*<.001).

### Research Question #4: TDF Domains

Table 8 shows the number of times the groups used each TDF domain in their tweets. In both cases, the TDF domain *skills* was the most used, although it appeared to be more frequently used by dietitians than by the public (612/1417, 43.2% vs 529/1469, 36.0%; *P*<.001). Other differences were also revealed between groups. Additionally, in both groups, it was found that the environmental context and resources, and more specifically, the pandemic, acted as important factors in the adoption of specific behaviors such as exercising at home or modifying a diet.

Table 9 depicts the most and least referenced domains per theme. This puts into light the TDF domains mostly associated with each theme or thematic category and could potentially be used to encourage behaviors related to the said themes or categories. Generally, themes related to weight management (weight loss, body appearance, diets, and dietary patterns) were associated with goals, and environmental context and resources. Food- and supplement-related themes (cooking and recipes, immune health, specific foods, alcohol consumption, nutrients, and supplements) were mostly associated with knowledge, skills, and environmental context and resources. Furthermore, these same 3 TDF domains (knowledge, skills, and environmental context and resources) were equally associated with themes about the food and health care systems (food support and food system, grocery, and health care system). Finally, lifestyle habit–related themes (food overconsumption, food tips and recommendations, food changes, and other lifestyle habits) were more commonly paired with skills, environmental context and resources, and behavioral regulation. Thus, for instance, goal setting could be considered when trying to lose weight or skills development could be implemented to encourage cooking.

**Table 8:** Comparison of the frequency of Theoretical Domains Framework domains between groups.

Domain	Dietitian group (N=1417), n (%)	Public group (N=1469), n (%)	*P* value
Knowledge	576 (40.7)	265 (18.0)	<.001
Skills	612 (43.2)	529 (36.0)	<.001
Social and professional role and identity	123 (8.7)	17 (1.2)	<.001
Beliefs about capabilities	100 (7.1)	114 (7.8)	.47
Optimism	121 (8.5)	106 (7.2)	.19
Beliefs about consequences	354 (25.0)	306 (20.6)	.008
Reinforcement	303 (21.4)	375 (25.5)	.009
Intentions	43 (3.0)	64 (4.4)	.06
Goals	61 (4.3)	290 (19.7)	<.001
Memory, attention, and decision processes	105 (7.4)	49 (3.3)	<.001
Environmental context and resources	471 (33.2)	482 (32.8)	.81
Social influences	50 (3.5)	41 (2.8)	.26
Emotion	130 (9.2)	61 (4.2)	<.001
Behavioral regulation	246 (17.4)	465 (31.7)	<.001

**Table 9:** The frequency of Theoretical Domains Framework domains for individual themes.

Theme	Most frequent domain	Frequency, n (%)	Least frequent domain	Frequency, n (%)
Weight loss (N=130)	Goals	59 (45.4)	Memory, attention and decision processes, and emotion	3 (2.3)
Cooking and recipes (N=429)	Skills	343 (80.0)	Social and professional role and identity	5 (1.2)
Immune health (N=264)	Knowledge	200 (75.8)	Intentions	2 (0.8)
Food support and food system (N=265)	Environmental context and resources	153 (57.7)	Social influences	5 (1.9)
Specific foods (N=665)	Environmental context and resources	273 (41.1)	Social and professional role and identity, and emotion	6 (0.9)
Alcohol consumption (N=105)	Environmental context and resources	60 (57.1)	Optimism and social influences	2 (1.9)
Nutrients and supplements (N=161)	Knowledge	105 (65.2)	Social and professional role and identity, and emotion	2 (1.2)
Food overconsumption (N=83)	Environmental context and resources	55 (66.3)	Social and professional role and identity	1 (1.2)
Food tips and recommendations (N=361)	Skills	258 (71.5)	Intentions	8 (2.2)
Food changes (N=322)	Environmental context and resources	232 (72.1)	Social and professional role and identity	9 (2.8)
Body appearance (N=153)	Environmental context and resources	69 (45.1)	Social and professional role and identity	3 (2.0)
Diets and dietary patterns (N=533)	Environmental context and resources	326 (61.2)	Social and professional role and identity, and memory, attention, and decision processes	4 (0.8)
Other lifestyle habits (N=712)	Behavioral regulation	389 (54.6)	Social and professional role and identity	15 (2.1)
Grocery (N=339)	Skills	205 (60.5)	Social influences	7 (2.1)
Health care system (N=232)	Knowledge	101 (43.5)	Social influences	4 (1.7)

## Discussion

### Principal Findings

This study found differences between dietitians’ tweets and the public’s tweets about the themes they discuss, the engagement they received from users, the TDF domains they used, and their content accuracy.

Differences about more frequently discussed themes were found between groups. Grocery was the most addressed theme by dietitians. Immune health, food support and food system, food tips and recommendations, grocery, and health care system were also more frequent in this group than in the public group. Conversely, the public group was mostly interested in discussing diets and dietary patterns, while weight loss, specific foods, alcohol consumption, food overconsumption, diets and dietary patterns, and other lifestyle habits emerged as more salient themes in this group than in the dietitian group.

Indeed, concerns have been raised by the population over grocery store safety practices, grocery bills, and an altered food supply [63], with the latter even leading to food shortages and elevated prices [64]. Furthermore, nutrition-induced immunity has been extensively addressed in the literature since the onset of the pandemic. However, online and social media posts on “immunity boosting” have contributed to the spread of misinformation and disinformation [9,26]. It is therefore possible that these were considered by dietitians as 2 areas of concern needing to be addressed by health professionals. Furthermore, results from Twitter users do not come as a surprise as these themes have been subjects of concern in the population during the pandemic. For instance, a survey conducted among adults from the Canadian province of Quebec revealed that weight-related concerns increased in 43% of participants [15]. Moreover, changes in dietary patterns and choices as well as alcohol consumption during the pandemic have been reported in different studies [13,65], just like modifications in weight and physical activity [66,67].

Moreover, as could be expected, thematic analyses between waves demonstrated that most of the discussions on nutrition and COVID-19 took place during the first wave, but more so in the case of dietitians. These results are supported by other studies. For instance, between January and October 2020, Google Search trends about COVID-19 and wine, ginger, 5G network spread, and the sun generally peaked in March and April 2020 [68]. Similarly, Chinese social media posts on COVID-19 misinformation peaked in February and March 2020 before slowly decreasing through May 2020 [69]. The disease novelty, concerns, sudden interest, anxiety, need for information, and necessity to adapt to an out-of-ordinary situation possibly drove the conversation.

In addition, contrary to our expectations, no general thematic popularity was revealed across the 3 types of user engagement reactions, as only the number of likes differed between groups according to the theme. As opposed to the study by Hand et al [37], where individual RDs did not receive retweets of their heart failure–related tweets, the retweet count for dietitians was fairly elevated in this study. Dietitians constantly received more retweets and the public received more replies. Retweet behavior could partly be explained by the dietitians’ authoritativeness, associated with their accurate knowledge of food and nutrition [70]. Moreover, although follower count could potentially influence dietitians’ higher retweet values and high variability [70], no difference in follower count was found between groups. Moreover, in the dietitian group, there was no association between retweet and follower values and only a weak association between like and follower values. Similarly, Harris et al [38] showed that the number of followers was not associated with retweets or likes in their study. Discrepancies in replies in their study could not be justified by any of the predictors analyzed. While the results are mixed, they are still promising considering that dietitians received more retweets, that retweet dissemination was exponential, and that where differences were found, dietitians received more likes than the public in all cases but one (ie, other lifestyle habits). Studies on the factors of engagement in nutrition-related tweets and differences in the types of reactions are warranted to optimize interest in dietitians’ tweets.

Contrary to other studies that have used the TDF to analyze specific aspects of nutrition or COVID-19, the model served a different purpose in this paper, as multiple nutrition and COVID-19–related behaviors were evaluated in tweets. Hence, all domains were addressed, suggesting that tweets could potentially contribute to behavior change. Additionally, differences were found between groups. However, in general, literature on the TDF mostly addressed the facilitators and barriers to the implementation of various behaviors by specific groups, which differs from how it was used in this study and renders the group comparison difficult. For instance, research on COVID-19 vaccine uptake has shown that themes related to the TDF domains of knowledge, beliefs about consequences, environmental context and resources, social influence, and emotion explain hesitancy [33], while facilitators have been found in beliefs about consequences [71]. Furthermore, another study found that 13 out of the 14 TDF domains explained nurses’ physical activity and eating behavior [72]. These factors compare to those in this study, which further implies that tweets could partly influence behavior. Nonetheless, barriers and facilitators are group and behavior dependent and might not apply in this context. Thus, studies on the barriers and facilitators to the implementation of specific nutrition-related behaviors (eg, grocery shopping habits) in times of a pandemic are warranted to determine how tweets should be phrased to influence behaviors.

Furthermore, a high proportion of tweets were considered not applicable for accuracy evaluation, which could be explained by the fact that Twitter is a means “to share quickly where one is, and what one is doing, thinking, or feeling” [73]. Therefore, especially in the public’s case, it still might not spontaneously be used to share verifiable facts and guidelines. This brings up the question as to whether Twitter represents the most useful or detrimental platform to seek health, nutrition, and pandemic-related information. However, for those tweets that were evaluated, as expected, a larger proportion of dietitians’ tweets about nutrition and COVID-19 were accurate compared with the public’s tweets. The higher quality and accuracy of dietitians’ blog posts compared with those of nondietitians has been shown before [74], although studies making these comparisons on social media are lacking. This study is one of the first to cast light on the difference in social media post accuracy between dietitians and the public.

### Practical Implications

Content accuracy results support the dietitians’ role in sharing reliable information on nutrition during a pandemic. Health and governmental agencies should make use of their valuable expertise during health crises, namely by identifying and allying with dietitians who are present and active on social media. This collaboration could also result in more sustained engagement not only in the COVID-19 and nutrition discourse on Twitter but also in other nutrition-related situations and conditions on the part of dietitians.

Moreover, differences in themes addressed by groups, engagement in the form of likes, and theme inaccuracy shed light on the themes that should be prioritized, further discussed, and made more engaging by dietitians to counter the potentially inaccurate tweets of the public. For instance, other lifestyle habits were more interesting to readers when addressed by the public, while weight loss had more inaccurate than accurate tweets. Characterizing the conversation on nutrition and COVID-19 is equally necessary to bring other health professionals to help dietitians in their work toward reducing misinformation and disinformation on Twitter.

Likewise, knowing the behavior change factors employed by each group helps in orienting social media interventions aiming at the adoption of favorable pandemic-related practices. It does so by prioritizing behavior change techniques associated with the most popular determinants (eg, skills), by further integrating ones that tend to be less used or ones recognized as facilitators and barriers of similar behaviors, and by considering the fact that a pandemic acts as a socioenvironmental factor that largely influences behavior.

Lastly, comparison of the frequency of tweets between waves demonstrated that most of the conversation on COVID-19 and nutrition happened during the first few months of the pandemic. Thus, efforts should be made early to counter misinformation and disinformation. Without giving support to a piece of false information, it becomes important to correct it as soon as it starts to spread widely [75,76]. This underlines the importance of being prepared by building timely social media interventions that will not overload readers with information and the importance of encouraging platforms, such as Twitter, to be ready to put in place countermeasures early during a crisis.

### Limitations

This study is not without limitations. First, although the methodology used to collect and validate tweets was rigorous, some of the keywords and hashtags were not specific to COVID-19 or nutrition, but were only related to it (eg, mask, disinfectant, and health). This resulted in a data collection that was possibly very sensitive but not specific enough. However, during coding, tweets were manually filtered to only keep those pertaining to the research theme. Hence, a lesser number of COVID-19 and nutrition-specific words should have been used to collect tweets. A keywords list should indeed be reviewed iteratively before initiating data collection [50]. Second, our use of the TDF differs from its prior use in research. Therefore, no similar methodology was available to inform our coding with the model, which could possibly be improved upon given the low initial kappa scores. For example, Griffith et al categorized tweets in a few themes before mapping these onto the TDF [33]. Third, the number of themes in the codebook and assigned to a given tweet should be limited to reduce the variability between coders. Fourth, the RD sample was potentially not representative of groups of dietitians outside of Canada and the United States. Similarly, although an efficient strategy was adopted to identify RDs, the use of the Dietitians of Canada Member Blogs list and the Nutrition Blog Network author directory potentially excluded a relatively high number of dietitians active on Twitter. Finally, it is possible that health professionals, including dietitians, were part of the public sample, which could have potentially influenced accuracy results. Nevertheless, we ensured that no dietitian from our sample was present in the public group.

### Conclusion

This study sheds light on the information sharing behaviors of RDs from Canada and the United States, and Twitter users in the COVID-19 and nutrition infodemic on Twitter. Differences were found in discussed themes, use of TDF domains, content accuracy, and generated user engagement. Studies and results like these are needed to support the role of practical, timely, and theory-informed social media interventions led by dietitians, as well as other health professionals specialized in their respective fields, for encouraging sound and evidence-based pandemic-related practices and behaviors.

## Abbreviations

RD: registered dietitian
TDF: Theoretical Domains Framework
